# Analysis of Patient Satisfaction through the Effect of Healthcare Spending on Waiting Times for Consultations and Operations

**DOI:** 10.3390/healthcare10071229

**Published:** 2022-06-30

**Authors:** José Manuel Santos-Jaén, María del Carmen Valls Martínez, Mercedes Palacios-Manzano, Mayra Soledad Grasso

**Affiliations:** 1 Department of Accounting and Finance, University of Murcia, 30100 Murcia, Spain; jmsj1@um.es (J.M.S.-J.); palacios@um.es (M.P.-M.); 2 Mediterranean Research Center on Economics and Sustainable Development, 04120 Almería, Spain; mayragrasso21@gmail.com; 3 Economics and Business Department, University of Almeria, 04120 Almería, Spain

**Keywords:** patient satisfaction, healthcare spending, healthcare quality, consultant waiting times, operation waiting times, gender perspective, partial least squares structural equation modelling (PLS-SEM)

## Abstract

In recent years, public authorities have invested large amounts of public money in trying to reduce waiting times for consultations and operations with the aim of improving the quality of the healthcare system. Our research aims to analyze the effect of these investments on patient satisfaction through the mediating relationship of waiting times for consultations and operations, as well as from a gender perspective. By studying a series of key indicators of the Spanish healthcare system and applying partial least squares structural equation modelling (PLS-SEM), the findings show that the model explains 12.10% of the variance in consultant waiting times, 51.90% in operation waiting times, and 27.00% in patient satisfaction. We found that increased public spending leads to increased patient satisfaction by reducing waiting times. However, no gender-based differences were found. The results provide exciting implications for theory and practice, indicating how policymakers can orient their strategies towards improving patient satisfaction.

## 1. Introduction

In recent years, governments in developed countries have invested huge amounts of money in improving the quality of their healthcare systems in order to increase patient satisfaction [1]. According to the World Health Organization, the countries that have invested the most in their healthcare systems in recent years are Sweden, Japan, Germany, and Norway [2].

However, it should be noted that these investments have decreased in recent years due to periods of economic recession [3]. In Spain, the evolution of public healthcare spending has been characterized by two circumstances:

1. The extraordinary development of Spanish public health care. Between 1984 and 2000, the number of public health accounts rose from 5800 million to 29,121 million.
2. Its progressive decentralization, culminating in the total territorialization of public health care in 2002 and the assumption of complete authority over their own healthcare systems by the different autonomous communities [4].

In line with the aforementioned circumstances, spending on the Spanish healthcare system totaled 115,458 million euros in 2019, representing 9.3% of the Gross Domestic Product (GDP) and an increase of 5% over 2018. 

Despite the large amount of money invested in the healthcare system, there is still a major problem concerning waiting lists for both consultations and surgery, as is the case in other public healthcare systems [5,6]. In Spain, the magnitude of this problem differs depending on the authority granted to each individual autonomous community by the government. For example, in 2012 the average waiting time for an ophthalmology consultation in the Balearic Islands was 163 days, whereas in the Basque Country, it was only 27 days. This is also the situation regarding waiting times for surgical interventions. For example, in 2014, the average waiting time for a cardiology intervention in the Canary Islands was 257 days, while in La Rioja it was only 35 days. These delays in access to healthcare services affect patients’ health [7] as well as the quality of the healthcare service, and consequently patient satisfaction [8,9]. In Spain, Law 14/1986 granted the autonomous communities complete authority regarding their individual healthcare systems [10]. This led each of them to organize their healthcare systems differently from the others, allocating varying amounts of economic and human resources, which accounts for the large differences in waiting times observed.

In theory, waiting lists occur when the demand for a given service exceeds the immediately available supply of that service. To resolve this situation, public authorities could allocate greater resources to their healthcare systems in order to improve the infrastructure and employ more personnel, thereby reducing waiting times. However, as Atalan [11] states, it is impossible to achieve positive results in the short term solely by increasing resources; more efficient management of these resources is also required. There is no doubt that delays in access time to the healthcare system can have serious consequences for patients, as this is associated with higher mortality rates [7]. These delays may be due to patient-related factors, such as age, gender, medical history, education, and income [7], as well as to inefficiencies in the healthcare system due to a lack of adequate infrastructure and/or healthcare personnel [12].

Therefore, accessibility to healthcare services has become essential for ensuring the quality of the healthcare system [13,14]. This has led governments in developed countries to launch a multitude of initiatives aimed at reducing waiting times, both for consultations and surgery [15], allocating huge amounts of public money to their healthcare systems [1].

It should also be considered that delays in access to hospital care lead to increased healthcare costs. This is because, in many situations where patients are not promptly attended to, their health deteriorates and they will require more medical attention. Thus, there is a correlation between delay times and healthcare costs [16]. In the same way, avoiding delays in operation waiting times is essential for providing timely, safe, and cost-effective health care [17]. 

The literature in this field, which includes no studies analyzing the effect of healthcare spending on reductions in waiting times, provides a number of solutions for reducing delays, such as increasing the supply of services, increasing the capacity of both the public and private sectors, and even increasing investments in telemedicine [18,19,20]. However, it is often difficult to implement these solutions due to limited available resources [18]. Patient satisfaction is a complex concept that can be summarized as the comparison between the expectations placed on a service and the perception of the service received [21]. Although this is a subjective concept and is therefore difficult to measure [21,22,23], it is important to study it as it is associated with improved clinical outcomes [24] and, consequently, with the quality of the healthcare system [10]. 

One of the main factors determining patients’ perceptions of the quality of a health service is their experience regarding access to health care [8,21,25,26,27]. Previous studies have shown a significant relationship between reductions in waiting times for health services, consultations, and interventions and increased patient satisfaction [3,8,28,29,30]. Similarly, previous studies have established that reducing perioperative delays can be an opportunity to improve the quality of care provided and, therefore, patient satisfaction [31,32]. One of the causes of delays in surgical procedures is delays in carrying out preoperative consultations, such as anesthesiology and cardiology [33]. Indeed, previous studies have established delays in patient preparation as being one of the main causes of delays in surgical procedures [17]. Thus, it is to be expected that the longer the waiting time for a consultation, the longer the waiting time for a surgical intervention. 

Previous studies have looked for a relationship between GDP per capita and patient satisfaction, comparing inequalities in healthcare spending between socioeconomic groups [34], and finding that citizens have a higher opinion of the healthcare systems in those regions with greater purchasing power. Furthermore, several studies have established that patients with greater purchasing power tend to feel higher levels of satisfaction as they can afford to use private health care, which is not as overcrowded as the public healthcare system [35,36]. However, other studies have concluded that there is no relationship between these two variables [3,10,37]. According to Valls Martínez et al. [10], a mediating relationship occurs when one variable interferes with two other related variables. This means that if the exogenous variable changes, it causes a change in the mediating variable, which in turn causes a change in the dependent variable. In this sense, as mentioned above, longer consultant waiting times lead to longer operation waiting times. Therefore, if increases in healthcare expenditure lead to reductions in consultant waiting times, it is to be expected that operation waiting times will also decrease indirectly. Likewise, increases in healthcare spending will indirectly improve patient satisfaction by reducing waiting times for both consultations and operations. This relationship between healthcare spending and patient satisfaction has been demonstrated in previous studies [10,35,37,38,39]. In addition, if consultant waiting times influence operation waiting times by delaying surgical interventions, then reductions in consultant waiting times will indirectly lead to increased patient satisfaction by reducing operation waiting times. 

Therefore, increases in healthcare spending will lead to greater patient satisfaction by reducing consultant waiting times and thereby reducing operation waiting times. 

Moreover, patients’ perception of and satisfaction with the healthcare system depends on their individual characteristics, such as age, gender, and social status [40,41,42]. Satisfaction with a service is divided into three elements: the structure, the processes, and the results obtained. Statistically significant differences between genders were found only for the processes element [40]. 

There are several reasons for believing that satisfaction with health care may depend on a person’s gender [43]. For example, it is more common for women to use healthcare services than men, as they often play a larger role in raising their children [41]. In accordance with the above and based on social role theory, the perception of health services may differ according to gender. However, this greater use of health services is not the only reason that can lead to a difference in perception between genders, which can also be influenced by factors such as health status, the different social constructions of illness, and social power relationships [42]. 

In recent years, researchers have become more interested in studying gender differences in levels of satisfaction with healthcare systems, although contradictory results have been found. On the one hand, these differences have been established and demonstrated by several studies [10,44,45,46]. On the other hand, however, other researchers have concluded that such differences do not exist [44,47]. The aforementioned demonstrates that patient satisfaction is a crucial element in evaluating quality of care [48]. For this reason, previous studies have investigated the relationship between patient satisfaction and expenditure from various perspectives [10,45]. However, our study introduces the use of consultant waiting time and operation waiting time as variables. We test how consultant waiting time influences operation waiting time and how both influence patient satisfaction. In addition, we verify how increased healthcare spending can reduce these waiting times. Finally, this study analyzes the effect of gender on these relationships. Therefore, the following research questions are proposed:

- Does consultant waiting time influence operation waiting time?
- Do waiting times for consultations and surgical procedures influence patient satisfaction?
- Does public healthcare expenditure influence waiting times and indirectly affect patient satisfaction?
- Does gender play a role in these relationships?

In order to answer these questions and for confirmation purposes, we developed a partial least squares structural equation modeling (PLS-SEM) on a sample of 272 observations by constructing a database based on the key indicators of the Spanish healthcare system supplied by the Spanish Ministry of Health, Consumer Affairs and Social Welfare.

This research makes an essential contribution to the literature by introducing the mediating effect of waiting times for consultations and operations into the relationship between public spending and patient satisfaction. Our findings may help public authorities find a solution to one of their main problems, namely, patient dissatisfaction due to long waiting lists for consultations and operations.

Following this introductory section, Section 2 shows the methodological aspects, Section 3 presents the results, Section 4 discusses these results and, finally, Section 5 presents the main conclusions.

## 2. Methodology

### 2.1. Sample and Data Collection

This study aims to determine the degree of patient satisfaction with the health system in Spain, as well as the factors that influence it. Therefore, based on the ideas described in the introduction, the following hypotheses were established:

**Hypothesis** **1** **(H1):** *GDP positively influences patient satisfaction.*

**Hypothesis** **2** **(H2):** *Consultant waiting times mediate the relationship between expenditure and operation waiting times.*

**Hypothesis** **3** **(H3):** *Consultant waiting times mediate the relationship between expenditure and patient satisfaction.*

**Hypothesis** **4** **(H4):** *Operation waiting times mediate the relationship between expenditure and patient satisfaction.*

**Hypothesis** **5** **(H5):** *Operation waiting times mediate the relationship between consultant waiting times and patient satisfaction.*

**Hypothesis** **6** **(H6):** *Consultant waiting times and operation waiting times sequentially mediate the relationship between expenditure and patient satisfaction.*

**Hypothesis** **7** **(H7):** *The relationship between consultant waiting times and patient satisfaction differs significantly between genders.*

**Hypothesis** **8** **(H8).** *The relationship between operation waiting times and patient satisfaction differs significantly between genders.*

**Figure 1:**
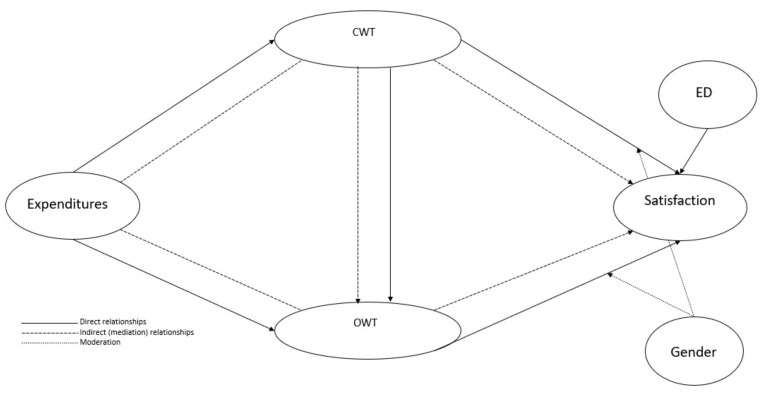
Conceptual model and hypotheses.

Figure 1 shows the conceptual model and the hypotheses developed in this study.

To carry out the study, a database was compiled, which contained healthcare expenditure and waiting times for consultations and surgical operations, as well as patient satisfaction with the public healthcare system. The data were compiled from the website of the Spanish Ministry of Health, Consumer Affairs and Social Welfare, which includes a section called Key Indicators of the National Health System (http://inclasns.msssi.es/, accessed on 15 April 2022). This provides, disaggregated by the 17 autonomous communities and the 2 autonomous cities that make up Spain, a list of indicators selected from the thousands of data available in the National Health System Information System from data sources managed by other official bodies. These are considered to be the most relevant indicators for understanding the health of citizens, the functioning of the public health system, and the factors that influence health [46]. Therefore, we worked with secondary data.

### 2.2. Variables

For all variables, we assumed a defining relationship between the variables and their indicators because of how the data were obtained. For this reason, we consider all variables as composite variables [49]. In PLS-SEM, the composites can be of two types: Mode A (correlation weights), in which the component indicators are expected to be correlated, and Mode B (regression weights), in which the indicators are expected to be uncorrelated [50]. Table 1 shows the definition and composition of the variables.

Expenditure (EXP) was estimated in Mode B because no correlation between the indicators was expected [51]. Spending may increase on some items and decrease on others. This variable comprised six indicators that measure the destination of Spanish healthcare spending. In Spain, each autonomous community has complete authority over its healthcare system. Therefore, healthcare spending is established in the general budgets of these autonomous communities, independently of each other.

Consultant waiting times (CWT) and operation waiting times (OWT) were estimated in Mode A because a correlation between the indicators was expected [51]. The delay in time for patient access to consultations and operations was generalized for all medical specialties. CWT was made up of nine indicators that measure the average waiting time, expressed in days, for a first consultation in different medical specialties of the Spanish health system. OWT was made up of six indicators that measure the average waiting time, expressed in days, for a surgical intervention in the different medical specialties of the Spanish health system.

Satisfaction (SAS) was estimated in Mode B as there is no correlation between the indicators [51]. Patients may be satisfied with the functioning of one part of the healthcare system and dissatisfied with another. This variable was made up of three indicators that measure the degree of patient satisfaction with the Spanish healthcare system using the average of the satisfaction ratings collected on a Likert scale from 1 (very dissatisfied) to 10 (totally satisfied).

As a control variable, we used the Economic Driver (ED) variable, measured as the Gross Domestic Product (GDP) per capita [10].

### 2.3. Analytical Procedure

We developed a partial least squares structural equation modelling (PLS-SEM) to ensure the quality of the results obtained in this explanatory and predictive study. Our model consisted of two type A composites and two type B composites [52]. Another reason for choosing this method is that PLS-SEM does not require large samples or samples with a specific distribution [53]. Finally, PLS-SEM is particularly suitable for the study of moderations between variables through multigroup analysis (MGA) [54], and also for when the structural model is complex and includes many constructs, indicators, and/or model relationships [55].

Our study was conducted from the year 2012 to 2019. As the indicators of satisfaction are disaggregated by gender and the autonomous cities were excluded, the final sample comprised 272 observations (136 by men and 136 by women). 

The statistical power of the study was tested using the G*Power 3.1.9.2 software, Franz Faul, University of Kiel, Kiel, Germany [56]. Assuming a significance level of 5%, a statistical power of 80%, six indicators in the formative construct with the highest number of indicators, and an effect size of 0.15, we carried out an a priori analysis. Based on the results obtained, we would need 98 observations to be able to validate the effects resulting from this research [57]. Therefore, the sample size is adequate.

By using the SmartPLS 3.3.3 software, SmartPLS GmbH, Boenningstedt, Germany [58], the hypotheses set out in the proposed model were tested. For this purpose, 10,000 samples were taken in this study using the bootstrap method [59].

## 3. Results

The results obtained for the proposed model are presented below. We first evaluate the measurement model, followed by the structural model, with the analysis of the possible existence of mediation. Finally, we discuss the possible presence of moderation based on the patient’s gender.

### 3.1. Descriptive Analysis

Table 2 shows the descriptive statistics of the indicators that make up the latent variables. In the case of patient satisfaction, the total results are shown as well as the results disaggregated by gender. There were no gender-based differences in the other variables. As can be seen, the satisfaction of men was higher than that of women in terms of the functioning of the healthcare system and the information they receive. On the contrary, when analyzing satisfaction regarding follow-up care and the doctor’s knowledge of a patient’s medical history, women presented a higher level of satisfaction compared to men.

### 3.2. Measurement Model

#### 3.2.1. Mode A Composites

Latent variables set as Mode A composites (CWT and OWT) were analyzed to test the reliability of individual items, construct reliability, convergent validity, and discriminant validity (see Table 3). 

According to Hair et al. [60], the first step in evaluating reflective composites is to analyze the reliability of the items by examining the standardized factor loadings, which should return a value greater than 0.7. This was the case in our model. In the second step, the construct reliability was tested with Cronbach’s alpha, composite reliability, and Dijkstra and Henseler’s rho ratio. The reliability of all the constructs was proven, as the results returned values greater than 0.7 [61,62]. In addition, the findings showed that the average variance extracted (AVE) was greater than 0.5 for all variables [50]. This demonstrates the adequate convergent validity of the model. 

Discriminant validity was analyzed using the Fornell–Larcker criterion and the heterotrait–monotrait ratio of correlations (HTMT). The findings are shown in Table 4. The Fornell–Larcker criterion was satisfied as the correlations between each pair of constructs did not exceed the square root of the AVE of each of the constructs [63]. Moreover, the level of the HTMT was below the established maximum value of 0.85 [64]. Therefore, based on the results obtained, we may conclude that the discriminant validity is satisfactory. 

#### 3.2.2. Composites Mode B

The analysis of formative composites requires the study of possible collinearity problems, as well as the sign, magnitude, and significance of their weights. Since there were differences in the degree of satisfaction between men and women, the results will be displayed in a disaggregated form. The results are shown in Table 5.

As can be seen, all the values of the Variance Inflation Factor (VIF) were below the maximum reasonable value established in 3.3. Based on these results, collinearity problems can be ruled out.

When the weights, which provide information on the contribution of each indicator to its latent variable, are non-significant, they can be maintained if the loadings are significant or greater than 0.5 [55], which was the case for the indicators of our model.

### 3.3. Structural Model Analysis

The structural model analysis began with the examination of collinearity between constructs. As in the previous section, we expected to obtain a value lower than 3.3, albeit for the constructs, in this case [55]. As can be seen in Table 6, the VIF ranged from 1.000 to 1.877, so we can discard the existence of collinearity problems between variables. Table 6 shows the results for the global model; Appendix A and Appendix B show the results according to gender.

To analyze the sign, magnitude, and statistical significance of the path of the coefficients, a one-tailed bootstrapping test with 10,000 replications was applied. The findings showed that expenditure had a significant negative effect on CWT (β = −0.352 ***). Likewise, expenditure had a significant negative effect on OWT (β = −0.331 ***). On the other hand, CWT had a significant positive effect on OWT (β = 0.537 ***). However, the effect on satisfaction was non-significant (β = −0.122 ns). Likewise, the influence of OWT on satisfaction was negative and significant (β = −0.461 **). Finally, the results suggested that ED, as a control variable, had a positive and significant effect on satisfaction (β = 0.129 *), thereby confirming H1.

After studying the direct effects, the existence of possible mediating effects was analyzed. The findings showed that CWT mediated the impact of expenditure on OWT (β = −0.189 ***) and satisfaction (β = 0.043 ns), supporting H2 but not H3. In the first case, as the direct effect was also significant, mediation was partial and complementary. In the second case, although there was no direct effect, mediation was also partial because there was a sequential indirect effect of expenditure on satisfaction through CWT and OWT (β = 0.087 **), thereby supporting H6. However, the indirect effect of expenditure on satisfaction through OWT was significant (β = 0.153 **), thereby supporting H4. Finally, the indirect effect of CWT on satisfaction through OWT was negative and significant (β = −0.247 **), thereby supporting H5. The direct effect was also negative and significant, which indicates that the mediation was partial and complementary.

With respect to the size of the indirect effect, measured through the variance accounted for (VAF) [59], the indirect effect of expenditure on satisfaction was 100%, with 54.15% through OWT, 15.14% through CWT (non-significant), and an additional 30.71%, sequentially. Similarly, the indirect effect of expenditure on OWT was about 36.34% of the total effect through CWT. Finally, the indirect effect of CWT on satisfaction was about 66.93% of the total effect through OWT. 

The measurement of the explanatory capacity of the model was performed using the coefficient of determination (R^2^). R^2^ shows, through the predicting variables of an endogenous construct, how these can explain the variance [59]. The minimum expected value was 0.1 [65]. The findings showed that the model explained 12.10% of the variance in CWT, 51.90% in OWT, and 27.00% in satisfaction. Therefore, the model had good explanatory power, especially in the case of OWT [66].

According to Cohen [57], f^2^ is used to measure the size of the effect, i.e., the degree to which an exogenous construct helps explain a given endogenous construct in terms of R^2^. If the value is less than 0.02, it is considered that there is no effect, which only happened for CWT with satisfaction; hence, the path was non-significant. 

**Figure 2:**
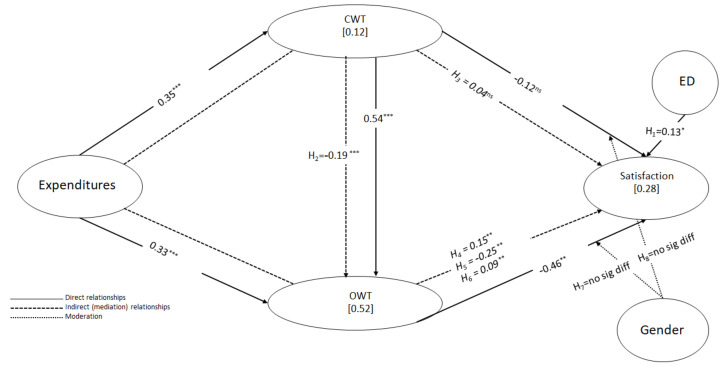
Whole model results. *: *p* < 0.05; **: *p* < 0.01; ***: *p* < 0.001; ^ns^ not significant.

Figure 2 shows the standardized path coefficients and R^2^. The standardized path coefficients explain the size of the contribution of the predictor variables towards the variance of the endogenous variables [67]. In addition, the R^2^ for each of the endogenous variables is also displayed and shows the variance explained by the variables that predict the endogenous variable. 

### 3.4. Further Analysis

The predictive relevance of the endogenous variables was tested using the blindfolding procedure, with the results showing that all Q^2^ values were greater than zero [68]. The blindfolding Q^2^ index is shown in Table 5.

Finally, the quality of the model was verified using the standardized root mean square residual (SRMR). The value obtained was 0.074, which was below the maximum required value of 0.08 [69].

### 3.5. Moderation Analysis

In the last analysis, a three-step MICOM multigroup analysis procedure was carried out to analyze the invariance of the composite models [64,70]. In this way, we were able to check whether the effect of gender was restricted to the path coefficients of the structural model and not to the parameters of the measurement model [71]. Our objective was to confirm the existence of measurement invariance. 

Since the indicators used for both groups were the same, the first step, configuration invariance, was satisfied. As for compositional invariance, the results in Table 7 show that all *p* values were greater than 0.05, thus satisfying this step. Finally, the existence of equality of means and variances was also satisfied, since the *p* values were greater than 0.05 [10]. These results allowed us to carry out the MGA analysis.

To determine whether differences existed between the groups, a permutation test was performed, where a *p* value ≤ 0.05 suggests that the discrepancy between group path coefficients is statistically significant [72]. Likewise, to obtain an additional confidence analysis, a non-parametric Henseler’s MGA procedure was developed; a *p* value lower than 0.05 or higher than 0.95 indicates significant changes [73]. The results are not shown as they returned similar results to the permutation test. In addition, a Welch–Satterthwaite test and a parametric test were applied. The first was used to check whether the two populations had equal means. A *p* value lower than 0.05 would indicate this inequality of means [74,75]. The parametric test returned similar results. Therefore, the findings in Table 8 show that we could not extrapolate a moderating effect of the gender variable on any of the relationships analyzed. Therefore, gender had no direct effect, which led us to reject hypotheses H7 and H8.

Only the relationships involving the satisfaction variable were displayed, as this was the only variable disaggregated by gender.

## 4. Discussion

Based on data on the main key indicators of the Spanish National Health System provided by the Ministry of Health, 25 indicators comprising five variables were analyzed. The objective was to analyze the effect of increased health spending on patient satisfaction by reducing waiting times for consultations and operations. The PLS-SEM method was employed to validate the relationships of the model established and to check for the possible existence of differences for patient gender.

The results confirmed the influence of health expenditure on waiting times for consultations and operations. As healthcare spending increased, waiting times decreased, thus confirming the theoretical contributions that stress the need to invest in infrastructure and personnel in order to reduce waiting times [18,19,20]. Regarding waiting times for surgical interventions, the results showed an indirect effect of healthcare spending on OWT since, by reducing consultant waiting times (CWT), the time required to perform surgical interventions decreased. This derives from the positive and significant effect that CWT had on OWT, since the longer the consultant waiting time, the longer the time required to perform planned operations with maximum guarantees of success [17].

The findings also indicated that reduced waiting times (CWT and OWT) increased patient satisfaction. Since access to health care services is one of the main determinants of patient satisfaction [8,21,25,26,27], our results confirmed previous studies which demonstrated that reductions in waiting times positively affected patient satisfaction [3,8,28,29,30,31,32]. Although the direct effect of CWT on satisfaction was non-significant, the results showed that the total effect of CWT was significant and indirect, as it negatively and significantly influenced OWT. Therefore, the results showed that patient dissatisfaction occurred when delays in consultant waiting times led in turn to delays in surgical interventions. This clarification is included in the conclusions to facilitate understanding of the results.

Furthermore, our results also indicated that, in line with previous research, increased healthcare spending influenced patient satisfaction indirectly by reducing waiting times for consultations and operations [10,35,37,38,39]. Moreover, this research performed an in-depth analysis of the influence of healthcare expenditure on patient satisfaction by investigating the mediating effect of both consultant waiting times and operation waiting times simultaneously and sequentially. The results revealed that both CWT and OWT played important mediating roles in understanding the relationship between healthcare expenditure and patient satisfaction. In conclusion, the higher the expenditure, the greater the level of patient satisfaction due to reductions in waiting times.

The results showed how the control variable, GDP per capita, positively affected patient satisfaction. Furthermore, the results confirmed previous studies that concluded that patients’ purchasing power was positively related to satisfaction, as they were less dependent on the overburdened public health system [35,36]. Therefore, we do not agree with those authors who have claimed that no significant relationship exists between per capita income and patient satisfaction [3,10,37].

Finally, with respect to the possible effect of gender on patient satisfaction, our results did not confirm the existence of such a difference.

This article is not without its limitations, which could be used to establish future lines of research. This study only analyzed the Spanish health system, so the results obtained here cannot be extrapolated to other countries. Therefore, future studies could employ a similar model in other geographical areas. In this research, the patient satisfaction construct explained 28% of the variance. It would be interesting to include other variables in the model that form a part of patient satisfaction, such as their participation in the diagnostic process or the attention they receive from health personnel, among others. In addition, we consider that it would be interesting to include aspects such as patient mortality in the model in order to see to what extent it is affected by waiting times. Finally, the study analyzed data up to the year 2019, prior to the onset of the COVID-19 pandemic. When the data for the following years become available, it would be very interesting to conduct this study again and analyze the effect of the pandemic on the established model. Therefore, the main limitation of this study is the availability of data.

## 5. Conclusions

With this research, we contribute to filling the gap regarding the direct effect of healthcare spending on waiting times for consultations and surgical interventions, as well as the indirect effect on patient satisfaction. Therefore, this research makes important contributions to the theory and research on public health by integrating into the literature the roles that CWT and OWT play in the relationship between public health expenditure and patient satisfaction. 

This research also has important implications for policymakers since it has been shown that an efficient increase in public spending results in increased patient satisfaction, which is one of the main concerns of the public authorities [13]. Furthermore, by reducing waiting times for both consultations and operations, it is possible to reduce healthcare spending by preventing many patients’ health from deteriorating, which results in increases in treatment costs. Finally, and most importantly of all, it will also improve patients’ health and reduce mortality rates [7].

## Figures and Tables

**Table 1:** Variables used in the research.

**Expenditure (EXP)**	
EXP_1	Percentage of expenditure on specialized care services
EXP_2	Percentage of spending on primary care services
EXP_3	Percentage of expenditure on public–private contracts
EXP_4	Percentage of spending on intermediate consumption
EXP_5	Percentage of public health spending on staff remuneration for resident training
EXP_6	Public health spending per capita
**Consultant Waiting Time (CWT)**	
CWT_1	Waiting times for Gynecology consultations
CWT_2	Waiting times for Ophthalmology consultations
CWT_3	Waiting times for Traumatology consultations
CWT_4	Waiting times for Dermatology consultations
CWT_5	Otorhinolaryngology office waiting times
CWT_6	General surgery office waiting times
CWT_7	Waiting times for Urology consultations
CWT_8	Waiting times for Digestive System consultations
CWT_9	Waiting times for Cardiology consultations
**Operation Waiting Time (OWT)**	
OWT_1	Waiting times for Gynecology procedures
OWT_2	Waiting times for Ophthalmology procedures
OWT_3	Waiting times for Traumatology procedures
OWT_4	Waiting times for Dermatology procedures
OWT_5	Waiting times for Otolaryngology procedures
OWT_6	Waiting times for Cardiac surgery procedures
**Satisfaction (SAS)**	
SAS_1	Degree of citizen satisfaction with the functioning of the public health system.
SAS_2	Degree of citizen satisfaction with the family physician or pediatrician’s knowledge of the patient’s medical history, as well as any possible follow-up care.
SAS_3	Degree of citizen satisfaction with the information received from the specialist regarding their health problems.
**Economic Driver (ED)**	
ED1	Gross Domestic Product (GDP) per capita

**Table 2:** Descriptive statistics.

Variables	Mean	SD	Variables	Mean	SD
CWT			Satisfaction (Total)		
CWT_1	57.366	43.188	SAS_1	6.635	0.520
CWT_2	68.968	34.453	SAS_2	7.638	0.390
CWT_3	71.645	29.724	SAS_3	7.380	0.470
CWT_4	59.008	23.465	Satisfaction (Men)		
CWT_5	40.855	20.109	SAS_1	6.643	0.399
CWT_6	42.468	27.079	SAS_2	7.598	0.271
CWT_7	52.629	30.701	SAS_3	7.391	0.371
CWT_8	54.452	29.301	Satisfaction (Women)		
CWT_9	50.573	24.697	SAS_1	6.627	0.389
OWT			SAS_2	7.684	0.242
OWT_1	72.227	25.614	SAS_3	7.364	0.365
OWT_2	87.667	35.819	Economic driver (ED)		
OWT_3	114.107	47.078	ED_1	23.101	4.765
OWT_4	80.265	45.061			
OWT_5	57.254	28.053			
OWT_6	87.409	40.505			
Expenditure					
EXP_1	62.184	3.361			
EXP_2	13.976	1.636			
EXP_3	25.542	3.871			
EXP_4	3.649	0.841			
EXP_5	47.715	4.644			
EXP_6	1464.31	156.27			

Standard Deviations (SD) performed by bootstrapping procedure with 10,000 replications.

**Table 3:** Assessment of the measurement model. Estimated constructs in Mode A.

	Loading	*t*-Student ***	Q2	α	ρA	ρC	AVE
CWT			0.065	0.907	0.925	0.924	0.578
CWT_1	0.705	24.486	0.119				
CWT_2	0.829	33.925	0.039				
CWT_3	0.752	22.809	0.050				
CWT_4	0.806	30.042	0.090				
CWT_5	0.859	42.276	0.057				
CWT_6	0.486	7.238	0.034				
CWT_7	0.701	19.345	-0.003				
CWT_8	0.794	26.787	0.090				
CWT_9	0.843	21.615	0.107				
OWT			0.300	0.850	0.906	0.892	0.594
OWT_1	0.899	54.603	0.474				
OWT_2	0.889	85.233	0.451				
OWT_3	0.448	6.746	0.019				
OWT_4	0.801	21.466	0.316				
OWT_5	0.546	12.146	0.121				
OWT_6	0.907	64.808	0.418				

Significance performed by bootstrapping procedure with 10,000 replications. Q2: cross-validated redundancies index performed using a nine-step distance-blindfolding procedure. α: Cronbach’s alpha; ρA: Dijkstra and Henseler’s composite reliability; ρC: Jöreskog’s composite reliability; AVE: Average Variance Extracted; ***: all loadings are significant at the 0.001 level.

**Table 4:** Discriminant validity.

	CWT	OWT
** CWT **	** 0.760 **	* 0.676 *
** OWT **	0.653	** 0.770 **

HTMT ratio over the diagonal (italics). Fornell–Lacker criterion: square root of AVE in diagonal (bold) and construct correlations below the diagonal.

**Table 5:** Assessment of the measurement model. Estimated constructs in Mode B.

Variables	Weights	*t*	CI 2.5%	CI 97.5%	Loadings	VIF	Q^2^
**Expenditure**							
EXP_1	0.730 ***	4.177	0.367	1.049	0.707 ***	2.989	
EXP_2	0.130 ^ns^	0.631	−0.275	0.536	−0.500 ***	3.236	
EXP_3	0.344 *	2.169	0.009	0.628	0.621 ***	2.338	
EXP_4	0.463 ***	3.983	0.226	0.678	0.639 ***	2.151	
EXP_5	0.414 **	2.737	0.094	0.688	0.013 ^ns^	1.618	
EXP_6	0.229 ^ns^	1.842	−0.023	0.464	0.147 ***	1.581	
**Patient satisfaction (Total)**							0.038
SAS_1	1.238 ***	4.146	1.114	1.321	0.751 ***	1.547	0.131
SAS_2	−0.357 ***	2.249	−0.610	−0.036	−0.052 ^ns^	1.677	0.003
SAS_3	−0.557 ***	2.334	−0.868	−0.144	−0.093 ^ns^	1.760	0.014
**Patient satisfaction (Men)**							0.023
SAS_1	1.247 ***	2.65	−1.013	1.375	0.743 ***	1.676	0.105
SAS_2	−0.131 ^ns^	0.501	−0.680	0.374	0.110 *	1.914	0.008
SAS_3	−0.747 ^ns^	1.526	−1.167	1.182	−0.120 *	1.876	0.029
**Patient satisfaction (Women)**							0.030
SAS_1	1.174 ^ns^	1.550	−1.163	1.295	0.719 *	1.448	0.104
SAS_2	−0.528 ^ns^	1.337	−0.786	0.704	−0.217 *	1.597	0.001
SAS_3	−0.403 ^ns^	1.083	−0.717	0.755	−0.101 *	1.713	0.012

* *p* < 0.10; ** *p* < 0.05; *** *p* < 0.01; ns, not significant. Significance, t-statistic, and 95% Bias-Corrected Confidence Interval performed by bootstrapping procedure with 10,000 replications. VIF, Variance Inflation Factor.

**Table 6:** Assessment of the global structural model.

GLOBAL	Path	SD	T-Value	f^2^	95 CI		H	Supported
Direct effects						VIF		
Expenditure -> CWT	−0.352	0.050	7.032 ***	0.142	[−0.452; −0.287]	1.000		Yes
Expenditure -> OWT	−0.331	0.054	6.141 ***	0.201	[−0.423; −0.246]	1.142		Yes
CWT -> Satisfaction	−0.122	0.098	1.236 ^ns^	0.011	[−0.280; 0.045]	1.877		No
OWT -> Satisfaction	−0.461	0.143	3.228 **	0.168	[−0.590; −0.316]	1.745		Yes
CWT -> OWT	0.537	0.047	11.435 ***	0.529	[0.454; 0.609]	1.142		Yes
ED -> Satisfaction	0.129	0.073	1.759 *	0.020	[0.005; −0.245]	1.140	H1	Yes
Indirect effects						VAF		
*Individual indirect effects*								
Expenditure -> CWT -> OWT	−0.189	0.032	5.823 ***		[−0.252; −0.145]	36.34	H2	Yes
Expenditure -> CWT -> Satisfaction	0.043	0.036	1.176 ^ns^		[−0.017; 0.103]	15.14	H3	No
Expenditure -> OWT -> Satisfaction	0.153	0.054	2.804 **		[0.097; 0.213]	54.15	H4	Yes
CWT -> OWT -> Satisfaction	−0.247	0.079	3.113 **		[−0.335; −0.160]	66.93	H5	Yes
Expenditure -> CWT -> OWT-> Satisfaction	0.087	0.033	2.676 **		[0.053; 0.134]	30.71	H6	Yes
*Global indirect effects*								
Expenditure -> Satisfaction	0.282	0.077	3.677 ***		[0.231; 0.345]	100.00		
Expenditure -> OWT	−0.189	0.032	5.823 ***		[−0.252; −0.145]	36.34		
CWT -> Satisfaction	−0.247	0.079	3.113 **		[−0.335; −0.160]	66.93		
Total effect								
Expenditure -> Satisfaction	0.282	0.077	3.677 ***		[0.231; 0.345]			
Expenditure -> OWT	−0.52	0.041	12.611 ***		[−0.600; −0.465]			
CWT -> Satisfaction	−0.369	0.104	3.554 ***		[−0.481; −0.234]			
OWT -> Satisfaction	−0.461	0.143	3.228 **		[−0590; −0.316]			

R^2^ adjusted [95% CI in brackets]: CWT: 0.121 [0.079; 0.201]; OWT: 0.519 [0.461; 0.597]; satisfaction: 0.270 [0.209; 0.357]; standardized path values reported. SD: Standard Deviation; f^2^: size effect index, values greater than 0.02, 0.15, and 0.35 represent small, medium, and large effect sizes; 95CI: 95% Bias-Corrected Confidence Interval; VIF: inner model Variance Inflation Factors; VAF: Variance Accounted Formula × 100 represents the proportion mediated. Significance, Standard Deviations, and 95% Bias-Corrected CIs were performed after applying bootstrap resampling for 10,000 subsamples; *: *p* < 0.05; **: *p* < 0.01; ***: *p* < 0.001. Only those total effects that differed from the direct effects are shown.

**Table 7:** Results of invariance measurement.

	Configuration Invariance (Same Algorithms for Both Groups)	Compositional Invariance		P-permutation Values	Partial Measurement Invariance Established	Equal Mean Assessment				Equal Variance Assessment				
Construct		Correlation original	5.0%			Difference	CI 2.5%	CI 97.5%	Equal	Difference	CI 2.5%	CI 97.5%	Equal	Full Measurement Invariance Established
Expenses	Yes	1.000	0.760	1.000	Yes		−0.233	0.223			−0.407	0.369		
ED	Yes	1.000	1.000	0.148	Yes		−0.222	0.249			−0.263	0.303		
Satisfaction	Yes	0.942	0.835	0.314	Yes	0.125	−0.248	0.213	Yes	−0.095	−0.434	0.381	Yes	Yes
CWT	Yes	1.000	0.996	1.000	Yes		−0.218	0.227			−0.415	0.389		
OWT	Yes	1.000	0.995	0.996	Yes		−0.219	0.251			−0.346	0.356		

CI: Confidence Interval.

**Table 8:** Multigroup Analysis (MGA).

	PERMUTATION				TEST W-S		PARAMETRIC	
	Path Coefficients (Women)	Path Coefficients (Women)	Diff	*p* Value	T	*p* Value	T	*p* Value
ED -> Satisfaction	0.080	0.148	−0.068	0.650	0.397	0.346	0.397	0.346
CWT -> Satisfaction	−0.066	−0.137	0.070	0.728	0.328	0.372	0.328	0.372
OWT -> Satisfaction	−0.500	−0.463	−0.036	0.792	0.084	0.467	0.084	0.467

W-S; Welch–Satterthwaite test. Diff: differences between groups.

## Data Availability

The data with which this research was carried out can be obtained from the Spanish Ministry of Health, Consumer Affairs and Social Welfare website http://inclasns.msssi.es/, accessed on 15 April 2022.

## Appendix A

**Table A1:** Assessment of the structural model for men.

MEN	Path	SD	T-Value	f^2^	95 CI		H	Supported
Direct effects						VIF		
Expenses -> CWT	−0.352	0.050	7.032 ***	0.141	[−0.452; −0.287]	1.000		
Expenses -> OWT	−0.331	0.054	6.141 ***	0.201	[−0.423; −0.246]	1.142		
ED -> Satisfaction	0.08	0.12	0.667 ^ns^	0.008	[−0.103; −0.292]	1.14		
CWT -> Satisfaction	−0.066	0.165	0.399 ^ns^	0.003	[−0.356; 0.190]	1.878		
CWT -> OWT	0.537	0.047	11.435 ***	0.528	[0.454; 0.609]	1.142		
OWT -> Satisfaction	−0.500	0.258	1.937 *	0.199	[−0.693; −0.109]	1.743	H1	Yes
Indirect effects						VAF		
*Individual indirect effects*								
Expenses -> CWT -> OWT	−0.189	0.032	5.823 ***		[−0.252; −0.145]	36.34	H2	Yes
Expenses -> CWT -> Satisfaction	0.023	0.065	0.355 ^ns^		[−0.076; 0.137]	8.15	H3	No
CWT -> OWT -> Satisfaction	−0.268	0.14	1.910 *		[−0.406; −0.005]	80.24	H4	Yes
Expenses -> CWT -> OWT-> Satisfaction	0.094	0.058	1.621 ^ns^		[−0.020; 0.175]	33.33	H5	No
Expenses -> OWT -> Satisfaction	0.166	0.097	1.707 *		[0.048; 0.255]	58.86	H6	Yes
*Global indirect effects*								
Expenses -> Satisfaction	0.282	0.127	2.231 **		[0.231; 0.345]	100.00		
Expenses -> OWT	−0.189	0.032	5.823 ***		[−0.222; −0.132]	36.34		
CWT -> Satisfaction	−0.268	0.140	1.910 *		[−0.406; −0.005]	80.24		
Total effect								
Expenses -> Satisfaction	0.282	0.127	2.231 **		[0.231; 0.345]			
Expenses -> OWT	−0.520	0.041	12.611 ***		[−0.600; −0.465]			
CWT -> Satisfaction	−0.334	0.152	2.194 ***		[−0.515; −0.041]			
OWT -> Satisfaction	−0.500	0.258	1.937 *		[−0.693; −0.002]			

R^2^ adjusted [95% CI in brackets]: CWT: 0.117 [0.066; 0.244]; OWT: 0.515 [0.661; 0.244]; satisfaction: 0.265 [0.188; 0.408]; standardized path values reported. SD: Standard Deviation; f^2^: size effect index, values greater than 0.02, 0.15, and 0.35 represent small, medium, and large effect sizes; 95CI: 95% Bias-Corrected Confidence Interval; VIF: inner model Variance Inflation Factors; VAF: Variance Accounted Formula x 100 represents the proportion mediated. Significance, Standard Deviations, and 95% Bias-Corrected CIs were performed after applying bootstrap re-sampling for 10,000 subsamples; *: *p* < 0.05; **: *p* < 0.01; ***: *p* < 0.001. Only those total effects that differed from the direct effects are shown.

## Appendix B

**Table A2:** Assessment of the structural model for women.

WOMEN	Path	SD	T-Value	f^2^	95 CI		H	Supported
Direct effects						VIF		
Expenses -> CWT	−0.352	0.050	7.032 ***	0.142	[−0.452; −0.287]	1.000		
Expenses -> OWT	−0.331	0.054	6.141 ***	0.201	[−0.423; −0.246]	1.142		
ED -> Satisfaction	0.148	0.122	1.217 ^ns^	0.027	[−0.114; 0.301]	1.139		
CWT -> Satisfaction	−0.137	0.139	0.979 ^ns^	0.014	[−0.345; 0.118]	1.876		
CWT -> OWT	0.537	0.047	11.435 ***	0.529	[0.454; 0.609]	1.142		
OWT -> Satisfaction	−0.463	0.354	1.310 ^ns^	0.173	[−0.618; −0.569]	1.746	H1	No
Indirect effects						VAF		
*Individual indirect effects*								
Expenses -> CWT -> OWT	−0.189	0.032	5.823 ***		[−0.252; −0.145]	36.27	H2	Yes
Expenses -> CWT -> Satisfaction	0.048	0.054	0.894 ^ns^		[−0.045; 0.135]	16.61	H3	No
CWT -> OWT -> Satisfaction	−0.249	0.188	1.322 ^ns^		[−0.364; 0.283]	64.67	H4	No
Expenses -> CWT -> OWT-> Satisfaction	0.088	0.075	1.666 ^ns^		[−0.106; 0.156]	30.45	H5	No
Expenses -> OWT -> Satisfaction	0.153	0.129	1.188 ^ns^		[−0.215; 0.228]	52.94	H6	No
*Global indirect effects*								
Expenses -> Satisfaction	0.289	0.212	1.366 ^ns^		[−0.321; 0.381]	100.00		
Expenses -> OWT	−0.189	0.032	5.823 ***		[−0.222; −0.132]	36.27		
CWT -> Satisfaction	−0.249	0.188	1.322 ns		[−0.364; 0.283]	64.67		
Total effect								
Expenses -> Satisfaction	0.289	0.212	1.366 ^ns^		[−0.321; 0.381]			
Expenses -> OWT	−0.521	0.057	9.172 ***		[−0.638; −0.451]			
CWT -> Satisfaction	−0.385	0.247	1.558 ^ns^		[−0.536; 0.319]			
OWT -> Satisfaction	−0.463	0.354	1.310 ^ns^		[−0.618; 0.569]			

R^2^ adjusted [95% CI in brackets]: CWT: 0.124 [0.073; 0.249]; OWT: 0.523 [0.445; 0.638]; satisfaction: 0.281 [0.206; 0.421]; standardized path values reported. SD: Standard Deviation; f^2^: size effect index, values greater than 0.02, 0.15, and 0.35 represent small, medium, and large effect sizes; 95CI: 95% Bias-Corrected Confidence Interval; VIF: inner model Variance Inflation Factors; VAF: Variance Accounted Formula x 100 represents the proportion mediated. Significance, Standard Deviations, and 95% Bias-Corrected CIs were performed after applying bootstrap re-sampling for 10,000 subsamples; ***: *p* < 0.001. Only total effects that differed from direct effects are shown.
